# Exercise-primed extracellular vesicles improve cell-matrix adhesion and chondrocyte health

**DOI:** 10.21203/rs.3.rs-2958821/v1

**Published:** 2023-06-06

**Authors:** Hirotaka Iijima, Kai Wang, Ella D’Amico, Wan-Yee Tang, Renee J. Rogers, John M. Jakicic, Fabrisia Ambrosio

**Affiliations:** Institute for Advanced Research, Nagoya University, Nagoya, Japan;; Biomedical and Health Informatics Unit, Graduate School of Medicine, Nagoya University, Nagoya, Japan;; Department of Physical Medicine and Rehabilitation, University of Pittsburgh, Pittsburgh, PA; Discovery Center for Musculoskeletal Recovery, Schoen Adams Research Institute at Spaulding, Boston, MA;; Department of Physical Medicine & Rehabilitation, Harvard Medical School, Boston, MA; Department of Physical Medicine and Rehabilitation, University of Pittsburgh, Pittsburgh, PA; Department of Environmental and Occupational Health, University of Pittsburgh, Pittsburgh, PA; Department of Internal Medicine, Division of Physical Activity and Weight Management, University of Kansas Medical Center, Kansas City, KS; Department of Internal Medicine, Division of Physical Activity and Weight Management, University of Kansas Medical Center, Kansas City, KS; Department of Physical Medicine and Rehabilitation, University of Pittsburgh, Pittsburgh, PA;; Discovery Center for Musculoskeletal Recovery, Schoen Adams Research Institute at Spaulding, Boston, MA;; Department of Physical Medicine & Rehabilitation, Harvard Medical School, Boston, MA

**Keywords:** extracellular vesicles, exercise, microRNA regulatory network, cell-matrix interactions, aging

## Abstract

Extracellular vesicles (EVs) have been suggested to transmit the health-promoting effects of exercise throughout the body. Yet, the mechanisms by which beneficial information is transmitted from extracellular vesicles to recipient cells are poorly understood, precluding a holistic understanding of how exercise promotes cellular and tissue health. In this study, using articular cartilage as a model, we introduced a network medicine paradigm to simulate how exercise facilitates communication between circulating EVs and chondrocytes, the cells resident in articular cartilage. Using the archived small RNA-seq data of EV before and after aerobic exercise, microRNA regulatory network analysis based on network propagation inferred that circulating EVs activated by aerobic exercise perturb chondrocyte-matrix interactions and downstream cellular aging processes. Building on the mechanistic framework identified through computational analyses, follow up experimental studies interrogated the direct influence of exercise on EV-mediated chondrocyte-matrix interactions. We found that pathogenic matrix signaling in chondrocytes was abrogated in the presence of exercise-primed EVs, restoring a more youthful phenotype, as determined by chondrocyte morphological profiling and evaluation of chondrogenicity. Epigenetic reprograming of the gene encoding the longevity protein, α-Klotho, mediated these effects. These studies provide mechanistic evidence that exercise transduces rejuvenation signals to circulating EVs, endowing EVs with the capacity to ameliorate cellular health even in the presence of an unfavorable microenvironmental signals.

## Graphical Abstract

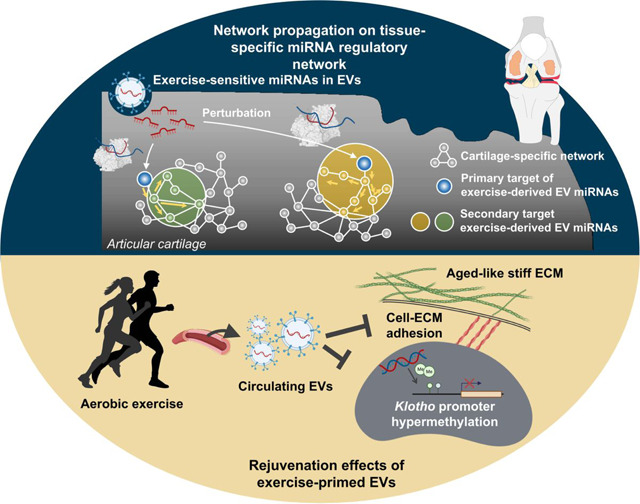

This study introduced network paradigms to simulate EV-mediated miRNA regulatory network perturbation in recipient articular chondrocyte and uncovered cellular rejuvenation effects induced by exercise-primed EVs *in vitro*. The observed rejuvenation effects was attributed, at least partly, to epigenetic reprogramming of the gene encoding the longevity factor, α-Klotho.

## INTRODUCTION

Extracellular vesicles (EVs) have emerged as a potent mechanism through which the beneficial effects of physical exercise are transmitted throughout the body^1^. EVs are nanovesicles that contain functional biomolecular cargoes, such as lipids, proteins, and nucleic acids^2^, which are highly responsive to physiological stressors and stimuli^3,4^. Several studies to date have shown that aerobic exercise modulates the bioactivity of circulating EVs, rendering EVs therapeutically beneficial in the context of disease such as cardiac ischemia-reperfusion injury, obesity, and type 2 diabetes mellitus^5,6^. However, the mechanism by which exercise alters the biological activity of circulating EVs and affects homeostasis of recipient cells remain unclear.

*In silico* computational approaches are useful for inferring the biological impact of EV biomolecules on recipient cells. This is particularly true for EV microRNAs (miRNAs), given that miRNAs can regulate post-transcriptional gene expression through complementary base pairing with mRNAs^7^. As such, the miRNA regulatory network has been used as a tool for dissecting the complex regulation by EVs on target biological processes^8,9^. Previous miRNA regulatory network analyses, however, have lacked consideration of the downstream secondary response of miRNA target genes on the global gene regulatory network. To simulate these downstream effects of EV miRNAs, network propagation is a powerful tool for elucidating the global transcriptional response induced by miRNAs. Network propagation on miRNA regulatory networks is further strengthened by consideration of tissue specificity given that the precise actions of genes are frequently dependent on the tissue in which the cells reside^10^. Indeed, human diseases result from the disordered interplay of tissue-specific processes^11^. The tissue-specific functional gene network clarifies biological functions of given genes to help identify disease driver genes that may exert important functional roles according to the tissue of interest

With this in mind, this study introduced network medicine paradigms to simulate EV-mediated miRNA regulatory network perturbation in recipient cells and uncovered cellular rejuvenation effects induced by exercise-primed EVs *in vitro*. This study simulated the effects of EVs on chondrocyte aging, a precursor for knee osteoarthritis (KOA). KOA represents a common age-related pathology that typically drives considerable functional declines. The well-established use of exercise for the prevention and treatment of KOA, as recommended by clinical practice guidelines^12^, further supports the use of this disease model to examine the downstream role of aerobic exercise-primed EVs.

## RESULTS

### Aerobic exercise upregulates miRNAs that modify chondrocyte-matrix adhesion processes

**Figure 1:**
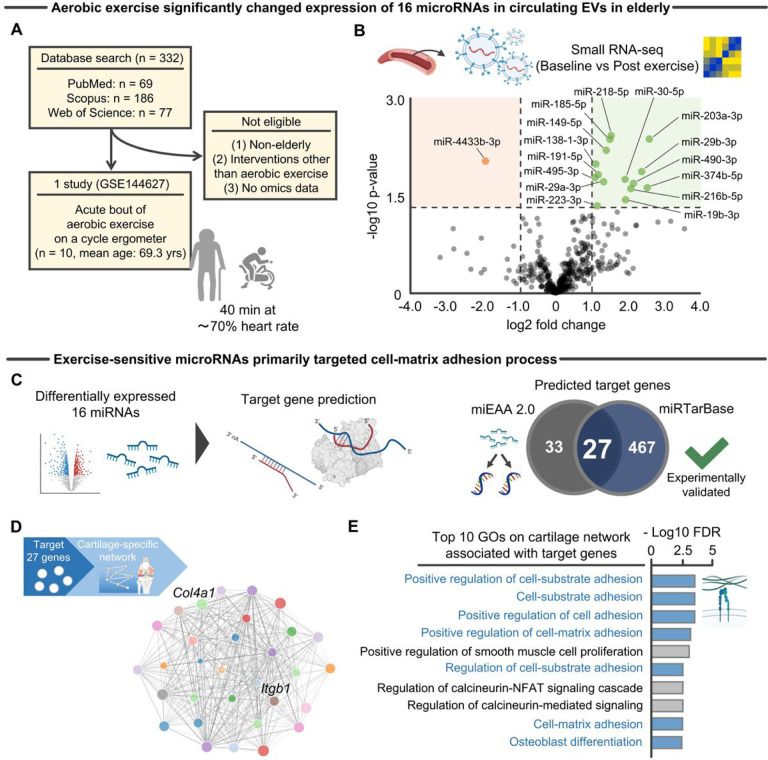
Small RNA-seq revealed that an acute bout of aerobic exercise significantly changed 16 EV miRNAs that primarily targeted the cell-matrix adhesion process **A,** An electronic database search yielded a total of 332 studies, of which 1 study (GSE144627)^3^ was finally included. The included study performed small RNA-seq from circulating EVs before and after an acute bout of aerobic exercise^3^. **B,** Small RNA-seq identified 16 differentially expressed miRNAs (−log2 p-value >1.3 and −log10 fold change >1.0). **C,** Target gene prediction of the differentially expressed 16 miRNAs predicted 27 target genes across two different database (miEAA 2.0 and miRTarBase). **D,** The 27 target genes were functionally connected on the cartilage-specific functional gene network. Network information was obtained using Humanbases software^24^. **E,** Cell-substrate adhesion process was the primary biological function significantly (FDR <0.05) associated with the 27 target genes, as indicated by GO enrichment analysis. Only the top 10 GOs were provided for illustration purposes. Portions of the figures were created with biorender.com. *Abbreviation: EVs, extracellular vesicles; FDR, false discovery ratio; GO, gene ontology*.

Most studies assessing the effects of exercise on EV biomolecules to date have focused on isolated miRNAs of interest, thereby precluding a holistic understanding of exercise-sensitive EV miRNAs and their downstream effects on recipient cells. To address this issue, we first performed a systematic literature search (Fig. 1A) and summarized EV miRNAs that are differentially regulated by exercise in older adults, as quantified by small RNA-seq. We identified only one study that performed small RNA-seq on circulating EVs in our target population, older adults, before and after exercise^3^. In that study, male participants (n = 10, mean age: 69.3 years old) completed an acute bout of aerobic exercise on a cycle ergometer for 40 minutes at under 70% of their maximum heart rate (Fig. 1A)^3^. Circulating EVs were then isolated from the serum collected before exercise, immediately after exercise, and after 3 hours of recovery. Participants were separated into two groups based on baseline physical activity level (sedentary vs trained; n = 5 per group). The authors demonstrated that aerobic exercise primarily changed miRNAs targeting the IGF-1 signaling pathway in an exercise-dependent manner^3^. To assess the global EV miRNA response to acute aerobic exercise, we accessed and evaluated the raw small RNA-seq data (GSE144627) from all 10 participants in the study. We then applied a filter as proposed by Chen et al^13^ in order to account for the bias caused by low expression count data. This filtering resulted in 442 miRNAs for analysis. Using this refined data set from 10 participants, we identified 16 differentially (log2 fold > 1.0, p-value < 0.05) expressed miRNAs (Fig. 1B). Of the identified miRNAs, 15 were significantly upregulated after aerobic exercise. Among these 15 miRNAs, two out of three miRNA29 family members, miR29a-3p and miR29b-3p, were significantly upregulated (Fig. 1B). The miRNA29 family is the “*master fibromiRNA regulator*” which targets at least 16 genes related to the extracellular matrix, including a large number of collagen isoforms and *Itgb1*^14^. This function is unique to miRNA29 given that no other miRNA targets more than 11 collagen genes^15^. In support of its unique function, the miRNA29 family has demonstrated anti-fibrotic actions in varied animal models of tissue fibrosis^16–18^, including chemically induced synovial fibrosis in murine knees^19^. The upregulated miR29a-3p and miR29b-3p after aerobic exercise is in line with the general understanding that exercise elicits anti-fibrotic effects^20^.

Next, we sought to determine the primary target genes of the 16 miRNAs. For this purpose, we used two different databases, miEAA.2.0^21^ and miRTarBase^22^, for target gene prediction (Fig. 1C). The integration of these two databases allows for determination of robust target genes that have been experimentally validated. The analyses identified 27 genes implicated in the process of cell-matrix adhesion, including *Itgb1* and *Col4a1*^23^, both of which are targets of the miR29 family^14^. Of note, when miR-4433b-3p, the only the miRNA downregulated by exercise, was used as an input, no target gene was consistently predicted. As such, all of the identified 27 target genes were the products of the remaining 15 miRNAs that were upregulated after aerobic exercise.

We then evaluated the biological function of these primary target genes. Given our focus on KOA, we generated a cartilage-specific functional gene network from the 27 target genes using HumanBases software^24^ (Fig. 1D). A functional relationship implies that two given genes participate in the same biological process in a specific tissue or functional context^10^. The tissue-specific functional gene network clarifies the biological function of genes in a tissue specific-manner^10^, thus helping identify biological processes that may exert a functional role in only the selected tissue(s)^11,25^. The generated cartilage-specific network revealed gene ontology (GO) terms related to integrin-mediated cell-matrix adhesion processes, including “*positive regulation of cell-substrate adhesion*” and “*cell-substrate adhesion*” (Fig. 1E). In articular cartilage, altered cell-matrix interactions initiate mechanotransduction processes, activating downstream signals that direct the transcription of genes involved in the regulation of cellular and tissue homeostasis^26^. Together, these *in silico* analyses inferred that miRNAs significantly upregulated after aerobic exercise primarily direct modification of chondrocyte-matrix adhesion and initiate downstream secondary effects.

#### Perturbation of the cartilage-specific miRNA regulatory network implicates exercise-primed EVs in the regulation of cartilage aging

**Figure 2:**
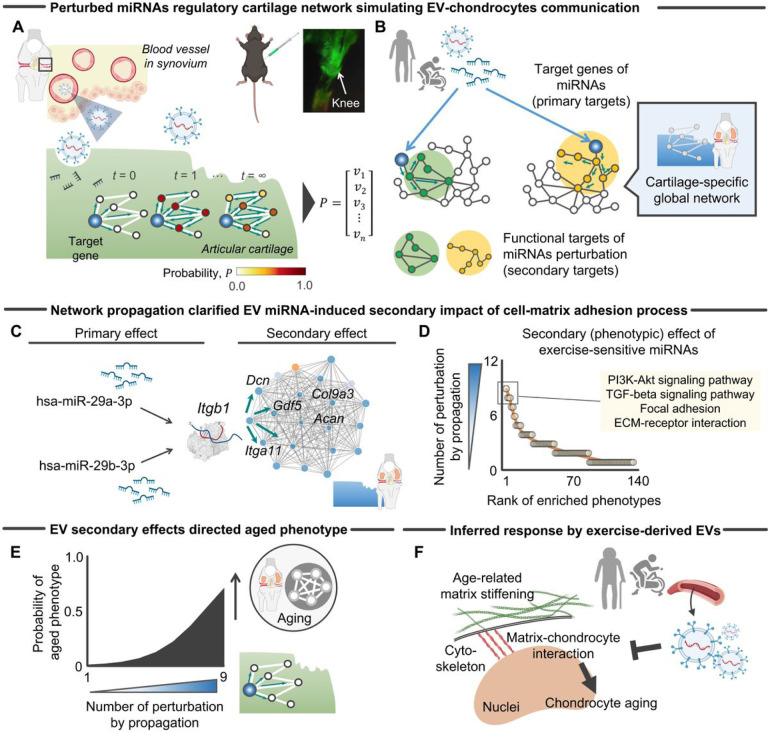
*In silico* perturbation of the miRNA regulatory network revealed that exercise-primed EV miRNAs primarily targeted signalings related to cartilage aging **A,** Schematic showing perturbation of miRNA regulatory network in articular cartilage that simulates EV-cartilage communication in the knee joint. This model is based on the assumption that circulating EVs are capable of penetration into joint cavity through the synovium, which is validated by the experiment showing tha localized fluorescence signal of systemically injected dyed EVs in murine knee joint cavity (see LI-COR image in the inset). Network propagation starting at each target gene was designed to infer downstream impacts of EV miRNAs on global cartilage functional network. **B,** Schematic showing the network propagation-based inference of phenotypic (downstream) targets of EV miRNAs. Network propagation explores the network vicinity of seeded genes (i.e., primary target of EV miRNAs) to infer their functions based on the premise that genes with similar functions tend to lie close to each other in the functional network. **C,** Example of a pertubed miRNA regulatory network of *Itgb1*, one primary target gene of EV miRNAs (has-miR29a-3p, has-miR-29b-3p). To infer phenotypic targets, 20 vicinity genes (20 highest probability scores) were used for the subsequent annotation of signaling pathways. **D,** Perturbation of miRNA regulatory network identified specific KEGG signaling pathways as a phenotypic target of exercise-primed EV miRNAs. **E,** The perturbed miRNA regulatory network frequently visited phenotypic targets related to cartilage aging that were previously identified^32^. **F,** Inferred chondrocyte responses induced by exercise-primed EVs. Portions of the figures were created with biorender.com. *Abbreviation: EVs, extracellular vesicles*.

To infer the downstream effects of the upregulated EV miRNAs on signaling cascade in articular cartilage (i.e., a downstream effect of chondrocyte-matrix adhesion), we implemented a network propagation approach to the cartilage-specific global functional gene network (Fig. 2A). Network propagation explores the network vicinity of genes of interest to study their functions based on the premise that nodes with similar functions tend to lie close to each other in the networks^27^. The network paradigm introduced here assumes an interaction between articular cartilage and circulating EVs that have penetrated into the joint cavity through the synovium, where intercellular gaps of the blood-joint-barrier (0.1 ~ 5.5 μm) are greater than EVs (~ 0.15 μm)^28^. To experimentally confirm the ability of circulating EVs to penetrate the joint cavity, labeled EVs were systemically administered to aged (21 months old) mice via intravenous injection. A localized fluorescence signal in the knee was observed 48 after systemic injection (see inset in Fig. 2A).

To simulate the EV-cartilage communication, we constructed a global cartilage-specific network using HumanBases software^24^. With this global network in hand, we then performed a random-walk-restart (RWR) algorithm^29^ to verify which gene nodes are most frequently visited on a random path in the cartilage-specific network. The aforementioned 27 target genes were used as seeded (starting) genes. Through this network medicine approach, we sought to determine the functional changes (i.e., KEGG pathways^30^) after miRNA perturbation (Fig. 2B). Figure 2C shows an example of *in silico* activation of *Itgb1*, a primary target of miR29a-3p and miR29b-3p^14^, on the network. RWR applied to a cartilage-specific network revealed that genes pseudo-activated (i.e., an affinity score > 0) by the 27 EV miRNAs target genes were highly associated with “*PI3K/Akt signaling*”, “*TGF-beta signaling*”, “*Focal adhesion*”, and “*ECM-receptor interaction*” (Fig. 2D), pathways that have been implicated in the pathogenesis of KOA^31^. To show the relationship between the functional targets and cartilage aging, we defined eight signaling pathways associated with cartilage aging according to mass-spectrometry proteomics data across the lifespan of male murine knee joints^32^. Logistic regression analysis revealed that functional targets with higher enrichment by network propagation displayed a significantly higher probability of an aged phenotype (Fig. 2E). These findings indicate that exercise-primed EV miRNAs secondarily regulate the cartilage aging process.

Studies have shown that aging changes biophysical properties of the ECM, a quintessential feature of aged tissue and a recently proposed hallmark of aging^33,34^. This is particularly true in articular cartilage, as evidenced by the findings that aged cartilage displays stiffness values that are more than twice the value of their young counterparts (E = ~ 50–100kPa in aged cartilage versus E = ~ 1–30kPa in young)^35^. A stiff ECM initiates, amplifies, and/or perpetuates aberrant ECM remodeling during aging^36^. This, in turn, disrupts chondrocyte function over time via mechanotransductive pathways^32,37–39^, eventually leading to KOA. Recent findings revealed that the age-related increased ECM stiffness drives healthy young murine chondrocytes to an aged phenotype via adhesion-mediated mechanotransduction and epigenetic regulation of the gene encoding the longevity protein, *Klotho*^32^. Chondrocyte phenotypic changes were accompanied by morphological changes, which was attributed, at least partly, to alterations in focal adhesion kinase levels^32,40^. Building from this previous work, our current findings from *in silico* analyses led us to ask two questions (Fig. 2F): (1) *do aerobic exercise-primed EVs counteract chondrocyte morphological changes as a surrogate measure of cell-matrix adhesion*, and (2) *can aerobic exercise-primed EVs counteract the compromised chondrocyte health induced by an aged-like (stiff) ECM?* The experiments that follow were designed to address these questions.

### Exercise-primed EVs overrode chondrocyte morphological alterations induced by a stiff ECM

**Figure 3:**
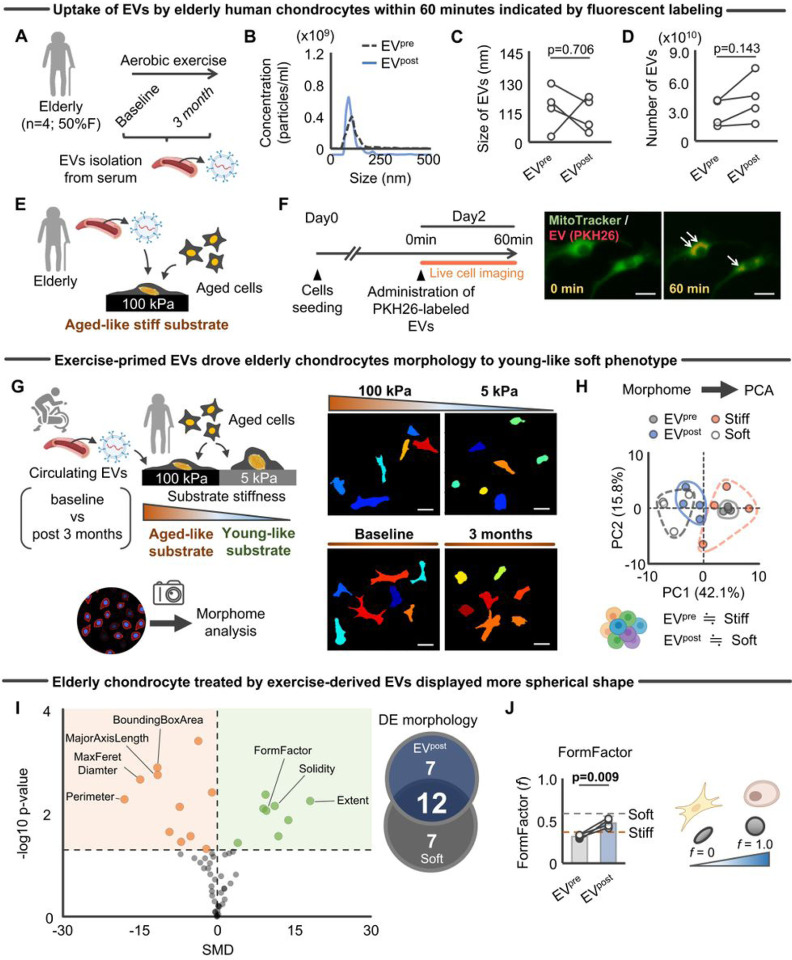
Exercise-primed EVs overrode chondrocyte morphometric alterations induced by aged-like stiff substrate **A.** Schematic showing the experimental protocol. Circulating EVs were isolated from serum before and after a 3-month aerobic exercise in healthy older adults (n = 4; 50% women). **B.** Representative data of nanoparticle tracking analysis (NTA) for EVs isolated before and after 3-month aerobic exercise. **C.** Change in size of EVs assessed by NTA (n = 4/group). **D.** Number of EVs across groups as assessed by nanoparticle tracking analysis (NTA; n = 4/group). **E.** Schematic showing the experimental protocol for co-culture experiment using the engineered *in vitro* system. Primary human chondrocytes isolated from older individuals were cultured on aged-like stiff (100kPa) substrate with EVs isolated before or after 3-month aerobic exercise. **F.** EV uptake by elderly chondrocytes. Representative snapshot from live cell image showing PKH26-labeled EV uptake by chondrocytes within 60 min. Scale bar: 50μm. **G,** Schematic showing the experimental protocol. Primary human chondrocytes isolated from older individuals were cultured on stiff (100kPa) or soft (5kPa) substrates. EV^pre^ or EV^post^ were then administered to cells cultured on a stiff substrate (100kPa) for evaluation of the morphological profile as assessed by CellProfiler^44^. Representative chondrocytic morphological images were obtained, which was reproducible in another set of images. Scale bar: 50μm. Cells were pseudocolored. **H,** PCA showing the separate clusters in chondrocyte morphology treated by EV^pre^ (gray circles) versus EV^post^ (blue circles) (n = 4/group). tChondrocytes treated with EV^post^ approximated those cultured on soft substrate (white circle). **I,** Exercise-primed EVs significantly changed 19 individual morphological variables compared to EVs isolated at baseline, in which the majority of morphological changes (12 variables) were consistent with those observed in chondrocytes cultured on young-like soft substrate (versus aged-like stiff substrate). **J,** Exercise-primed EVs significantly increased formfactor (i.e., higher cellular roundness) of aged chondrocytes (n = 4/group; quantified by immunofluorescence images with 60–110 cells per individual sample). Statistical analysis was performed using a two-tailed paired *t*-test (**C**, **D**) or Student *t*-test (**J**). Data are presented as means ± 95% confidence intervals. Portions of the figures were created with biorender.com. *Abbreviation: DE, differentially expressed; EVs, extracellular vesicles; EV*^*pre*^*, EV isolated at baseline; EV*^*post*^*, EV isolated after 3-month aerobic exercise; PCA, Principal component analysis*.

As a first step to address these questions, we conducted an exercise study in older individuals. Circulating EVs were isolated from older adults ages 65–85 years old (n = 4, 50% women) who completed 3-months of an aerobic exercise protocol that aimed to achieve a moderate-intensity based on perceived exertion (Fig. 3A). The exercise protocol was prescribed for 5 days per week and consisted of a combination of 1 supervised and 4 home-based, non-supervised sessions per week, which progressed from 70 minutes per week to 150 minutes per week across the intervention period. To facilitate engagement in the aerobic sessions, participants were provided with activity videos on a tablet that were developed by the study team. Exercises were taught in the supervised sessions and then the videos were used to facilitate the home-based, non-supervised sessions.

EVs were isolated using size-exclusion chromatography before and after 12 weeks of exercise exposure (EV^pre^ and EV^post^, respectively), using a previously described protocol^41^. EV purity was confirmed by western blotting for the presence of EV-specific markers, CD81, and the absence of non-EV marker, GM130 (**Figure S1**). As determined by NanoSight nanoparticle tracking analysis, average EV size and number were similar across the two groups (Fig. 3B–D), which is consistent with findings from previous exercise cohorts^42^.

EV^pre^ or EV^post^ were then co-cultured with aged human chondrocytes seeded onto a substrate that mimics the matrix stiffness of aged human cartilage (100kPa) (Fig. 3E)^35^. We used this experimental design to test the ability of the exercise-primed EVs to counteract age-related pathogenic mechanotransduction induced by aged-like stiff substrate. Prior to analysis, we confirmed the rapid uptake of fluorescently-labeled EVs by aged human chondrocytes (Fig. 3F). This is consistent with a previous study demonstrating that the uptake of circulating EVs by recipient C2C12 occurs within 60 minutes^43^.

To interrogate the ability of exercise-primed EVs to modulate chondrocyte-matirx adhesion processes, we assessed morphological alterations of chondrocytes cultured on a stiff substrate that were exposed to either EV^pre^ or EV^post^ (Fig. 3G). Specifically, we quantified chondrocyte morphology across 53 morphological variables using Cell Profiler software^44^. Cell Profiler simultaneously measures cell size, shape, intensity, and texture in a high-throughput manner. Figure 3G provides representative images of chondrocyte morphology across the different groups. We posited that exercise-primed EVs would interfere with the aged phenotype induced by a stiff matrix and drive a more youthful chondrocyte morpheme that resembles chondrocytes seeded on a soft substrate^32^. As such, the features of chondrocytes cultured on soft substrate were used as a reference value of a youthful profile.

Principal component analysis (PCA) analysis confirmed significant differences between the morphology of chondrocytes treated with EV^pre^ compared to those treated with EV^post^ (Fig. 3H). Notably, chondrocytes seeded on a stiff matrix treated with EV^post^ approximated cells cultured on a soft substrate (Fig. 3H), suggesting that the influence of EV^post^ on aged chondrocytes mimicks that of a soft (young-like) substrate. Figure 3I summarizes the changes in individual morphological features. Of the 19 morphometric variables significantly altered by EV^post^, the majority (12 [63.2%]) overlapped with the effect of a soft substrate (Fig. 3I). Among the individual morphogical variables of interest, we found that EV^post^ increased chondrocyte “formfactor” (Fig. 3J). “Formfactor” is a metric of cellular sphericity or roundness and was the feature previously identified in chondrocytes as highly sensitive to mechanical input^32^. On the other hand, aged chondrocytes treated with EV^pre^ did not display significant morphometric changes and more closely resembled untreated cells cultured on stiff substrate. Together, these results suggest that exercise-primed EVs can override the effects of a stiff matrix on chondrocyte morphology and support the network-based inference for the primary target of EV miRNAs (i.e., chondrocyte-matrix adhesion) (Fig. 1C–E).

### Exercise-primed EVs counteract the compromised chondrocyte integrity and Klotho promoter hypermethylation induced by a stiff ECM

**Figure 4:**
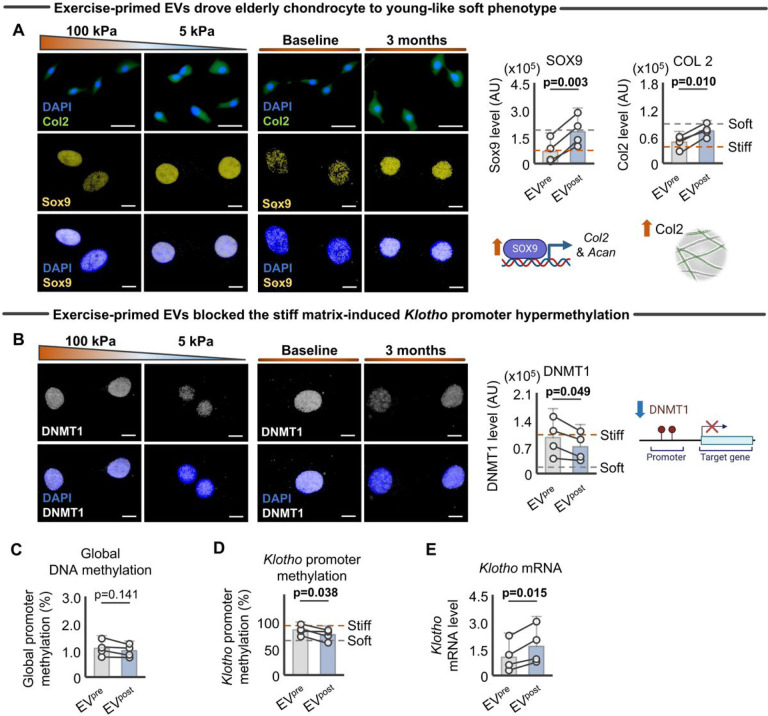
Exercise-primed EVs overrode compromised chondrocyte integrity and Klotho promoter hypermethylation induced by aged-like stiff substrate **A,** Exercise-primed EVs significantly increased type II collagen and Sox9 level (n = 4/group; quantified by immunofluorescence images with 20–100 cells per individual sample). Scale bar: 50μm (type II collagen) and 10μm (Sox9). **B,** Exercise-primed EVs significantly decreased DNMT1 (n = 4/group; quantified by immunofluorescence images with 15–20 cells per individual sample). **C,** Exercise-primed EVs did not significantly influence global DNA methylation in elderly chondrocytes (n = 4/group). **D,** Exercise-primed EVs significantly decreased *Klotho* promoter methylation in elderly chondrocytes (n = 4/group). **E,** Exercise-primed EVs significantly increased *Klotho* gene expression in elderly chondrocytes (n = 4/group). Statistical analyses were performed using a two-tailed paired *t*-test (**A-E**). Data are presented as means ± 95% confidence intervals. Portions of the figures were created with biorender.com. *Abbreviation: EVs, extracellular vesicles; EV*^*pre*^*, EV isolated at baseline; EV*^*post*^*, EV isolated after 3-month aerobic exercise*.

To next evaluate the ability of exercise-primed EVs to promote chondrocyte health, we repeated the above *in vitro* experiment, this time testing whether exercise-primed EVs can restore a more youthful chondrogenicity to aged cells. We found that aged chondrocytes treated with EV^post^ displayed increased type II collagen and Sox9 expression, two well-established markers of chondrocyte health (Fig. 4A). On the other hand, consistent with our morphometric analysis (Fig. 3G–J), aged chondrocytes treated with EV^pre^ displayed type II collagen and Sox9 levels comparable to cells without treatment (Fig. 4A). These findings further verify the network-based inference for the downstream target of EV miRNAs (i.e., cartilage aging process) (Fig. 2D–F).

Finally, we explored candidates that may drive the rejuvenating effect of EV^post^ on chondrocyte integrity. Our recent study demonstrated that a stiff substrate increases methylation of the *Klotho* promoter in chondrocytes^32^. Klotho is an upstream regulator of PI3K/Akt signaling^30^ and a so-called longevity protein that has been shown to attenuate the effects of aging on articular chondrocytes^32^. In response to a stiff substrate, transcriptional machinery is recruited to the *Klotho* promoter in murine chondrocytes, inhibiting the downstream anti-aging cascade^32^. One such transcriptional machinery is Dnmt1, an enzyme that catalyzes the transfer of methyl groups to CpG dinucleotides, usually repressing gene transcription by altering chromatin structure and blocking the access of transcription factors at the gene regulatory region^45^. Intriguingly, we found that elderly chondrocytes treated with EV^post^, but not those treated with EV^pre^, displayed significantly decreased Dnmt1 levels (Fig. 4B). While EV^post^-treated chondrocytes also displayed homogenous global DNA demethylation (Fig. 4C), the decreased Dnmt1 level was accompanied by decreased *Klotho* promoter methylation (Fig. 4D) and upregulated *Klotho* mRNA (Fig. 4E). These cumulative findings suggest that aerobic exercise-primed EVs can override the compromised chondrocyte integrity and *Klotho* promoter hypermethylation induced by a stiff ECM.

## DISCUSSION

While studies have evaluated EVs as a potent means to transpose the systemic benefits of exercise onto target cells, mechanistic evidence that exercise enhances the accumulation of health-promoting biomolecules in EVs in the context of aging has been lacking. This knowledge gap is particularly critical in the development of effective management for people with KOA given that currently available treatments focus exclusively on symptom management. As a first step to address this knowledge gap, this study introduced a network paradigm approach to infer functional targets of exercise-primed EVs in recipient aged chondrocytes. This approach revealed novel rejuvenating effects of exercise-primed EVs on aged chondrocytes (see graphical abstract; Fig. 5). Using the archived small RNA-seq data from EVs, the network medicine approaches inferred cell-matrix adhesion as a primary target of exercise-primed EVs, initiating downstream signals that direct cartilage aging. In support of this analytical prediction, we demonstrated that EVs isolated after aerobic exercise in older individuals overrode alterations in chondrocyte morphology and chondrogenicity induced by stiff ECM, a typical feature of aged articular cartilage^35^. The youthful phenotype induced following treatment with EVs that were isolated post-exercise was due, at least in part, to epigenetic reprogramming of *Klotho*. Collectively, the findings of this study suggest a mechanism by which circulating EVs released during/following chronic aerobic exercise may inhibit the aging process of distal joints.

The significance of the network paradigm introduced used in this study is the inference of downstream targets of EV miRNAs in a tissue-specific manner. This novel approach is based on the premise that the biological impact of EV miRNAs on target genes should propagate across the global network, not just across miRNA target genes, and that this impact is most effectively interpreted when considering a tissue-specific network. For example, miR29a-transfected chondrocytes displayed an upregulation of *Col2a1*^46^, even though *Col2a1* is not a direct target of miR29a^22^. This discordance highlights the limitation of miRNA target gene prediction to anticipate overall cellular responses. By implementing the network propagation approach on a tissue-specific network, we found that EV miRNAs that are modulated by exercise direct signaling pathways relating chondrocyte aging. PI3K/Akt signaling, which we found to be significantly inferred by network propagation as downstream of EV effects, has been implicated in the pathogenesis of human KOA^47^. The important role of this pathway in the pathogenesis of OA is further evidenced by *in vivo* studies showing that modulation of PI3K/Akt signaling attenuated pathological cartilage changes in the setting of post-traumatic KOA^48,49^.

Building on the *in silico* network-based inference, we demonstrated that exercise-primed EVs abrogated the deleterious effects of a stiff matrix on chondrocyte aging. It is well established that an age-related increase in matrix stiffness disrupts chondrocyte functionality via mechanotransductive pathways^37–39^. A recent study showed that a stiff matrix epigenetically represses the gene encoding the longevity factor, α-Klotho, resulting in chondrocyte dysfunction, a leading cause of OA^32^. This study provides evidence that exercise-primed EVs promote a youthful phenotype in aged chondrocytes, as evidenced by improved chondrogenicity and epigenetic reprograming of *Klotho*. This finding suggest that EV-based approaches may serve as a viable treatment for age-related KOA. Currently, no FDA-approved drug is available to target ECM mechanics by preventing or reversing tissue stiffening or interrupting the cellular response, although the field of mechanomedicine has been rapidly expanding^50^. In keeping with the spirit of this field, the identification of biomolecules that drive the exercise-induced epigenetic reprograming of *Klotho* is an interesting future work.

Although this study provides novel mechanistic insight into anti-aging effects of exercise, it has limitations. First, this study did not address *how* exercise alters EV bioactivity. It is reasonable to hypothesize that mechanical stress imposed by exercise alters the biogenesis and bioactivity of EVs, a hypothesis that is supported by studies demonstrating that mechanical stimulation of mesenchymal stem cells and lung epithelial cells promoted EV release and the expression of miRNAs in EVs^51,52^. Further research is needed to establish the mechanism by which exercise promotes the bioactivity of circulating EVs. Similarly, the tissue sources of circulating EVs released in response to exercise remain unclear and a worthwhile area of investigation. Second, the experimental study that compared EV^pre^ versus EV^post^ were based on a small number of participants in older adults, which may contribute to bias depending on participant characteristics. As such, the findings may not be generalizable to other age groups. Third, the exercise modality used in the current study was limited to aerobic exercise. As such, it is unclear whether the findings of this study are also applicable to other type of exercise (e.g., resistance exercise). Finally, this study focused exclusively on analyzing miRNAs to infer functional targets of exercise-primed EVs. We cannot rule out the possibility that other EVs cargos, such as proteins and/or lipids, may also play a role in counteracting aging phenotypes in chondrocytes. Such studies would be an interesting extension to our current finding that EVs have the potential to counteract the compromised chondrocyte integrity and *Klotho* promoter hypermethylation that results from an aged ECM.

## MATERIALS and METHODS

### Steps to ensure methodological rigor

While no established guideline is available for *in vitro* studies, we adapted Animal Research Reporting In Vivo Experiments (ARRIVE) essential 10^53^ and excluded randomization (item4) and experimental animals (item8). This study also followed reporting guideline proposed by Emmerich et al^54^. Where possible, power analysis from pilot data was done to select the number of animals needed for the study using Power and Sample Size Program (version 3.1.2; Vanderbilt University Medical Center, Nashville, TN)^55^. For example, sample size calculation estimated that four EVs before and after aerobic exercise in older adults were required to achieve statistical power of 0.8 based on the type II collagen expression. The treatments and image analyses of EVs isolated before and after exercise were conducted under the same conditions.

### Systematic literature search

We performed a systematic search to identify original articles investigating profiles of biomolecules in EVs (i.e., proteomics, RNA-sequencing, small RNA-sequencing, microarray) in older adults after aerobic exercise. Two independent reviewers (HI and ED) conducted an electronic search from the time of database inception to December 2021 using PubMed, Scopus, and Web of Science. A manual search was also performed using Google Scholar. The reference lists of relevant systematic reviews^1,6,42,56,57^ were manually searched. The same two reviewers then assessed the titles and abstracts of records identified using the prespecified eligibility criteria. Articles that passed this initial screening were further reviewed using their full manuscripts. Exclusion criteria included non-peer review journal, non-original article, language other than English, animal study, adults with young or middle-aged (i.e., < 65 years old), no aerobic exercise intervention, no circulating EVs, no omics data, full text not available, and raw data not available. Disagreements regarding manuscript inclusion between the two reviewers was discussed until a consensus was achieved. Finally, a citation search of the identified articles was performed using Web of Science to screen for potential additional articles. Identified records were then screened and reviewed using the same criteria. Customized Google spreadsheets were used by reviewers to assess and record eligibility.

### Differential expression analysis for archived EV small RNA-seq data

We accessed the archived small RNA-seq data (GSE144627) collected from the serum in older adults before exercise, immediately after exercise, and after 3 hours of recovery^3^. Raw count data was normalized by count per million (CPM), filterByExpr function, and Trimmed Mean Mvalue (TMM) using R/Bioconductor package edgeR with default parameters^58^. Differential expression analysis was performed for miRNAs with a normalized CPM value using R/Bioconductor package limma^59^. The Benjamini-Hochberg FDR control for multiple hypothesis testing was used to produce q-values.

### Target gene prediction of miRNAs

Primary target genes of differentially expressed miRNAs were determined using two different databases, miEAA.2.0^21^ and miRTarBase^22^. miEAA.2.0 is a updated web-based application that offers a variety of commonly applied statistical tests such as over-representation analysis and miRNA set enrichment analysis^21^. The target genes significantly predicted by mmiRAA.2.0 were then cross-checked by miRTarBase^22^ which is the experimentally validated miRNA-target interactions database^22^. Among the experimentally validated miRNAs, we have included genes with “strong” experimental evidence (i.e., genes supported by reporter assay, western blot, or qRT-PCR).

### Functional characterization of target genes using GO enrichment analysis on the cartilage-specific functional network

To determine the biological function of genes of interests, GO enrichment analaysis was performed on the cartilage-specific network constructed by HumanBase web tool^24^ with the target genes of interest used an input. Since the cartilage-specific network was established based on human tissue, all the gene symbols were translated into human gene symbols prior to the analysis.

### Gene perturbation on miRNA regulatory network using RWR

Cartilage-specific networks were constructed using HumanBase software^24^. On cartilage-specific global network, RWR was performed by R/Bioconductor package RandomWalkRestartMH^29^ with the each target gene used as a seeded node. RWR simulated a walker starting from one node or a set of nodes (seed nodes) in one network, and such walker randomly moved in the network to deliver probabilities on the seed nodes to other nodes. After iteratively reaching stability, the affinity score of all nodes in the given network to each target gene node were obtained. Cartilage-specific global network was constructed using a gold standard data set (i.e., already known gene interactions) downloaded from HumanBase software (https://hb.flatironinstitute.org/download)^24^.

### Functional characterization of in silico activated genes using pathway enrichment analysis

To determine the functional targets of gene perturbation on miRNA regulatory network, KEGG enrichment analysis was performed^30^. In this analysis, pseudo-activated (i.e., an affinity score > 0) genes after each RWR were used as an input.

### Determination of relationship between functional targets of gene perturbation and hallmarks of cartilage aging

Hallmarks of cartilage aging was defined as the eight KEGG pathways significantly changed over time according to mass-spectrometry proteomics data of articular cartilage across the lifespan of male murine knee joints^32^. Logistic regression analysis was performed to assess the relationship between functional targets of gene perturbation on miRNA regulatory network (i.e., number of perturbation by RWR; continuous) and hallmarks of cartilage aging (i.e., the presence of hallmarks of cartilage aging; 0: absence, 1: presence). The results were provided as a probability of being aged phenotype.

### LI-COR imaging after systemic injection of dyed EVs in aged mice

Aged (21 months) C57/BL6 male mice were obtained from the NIA Rodent Colony and Jackson Laboratories. Prior to inclusion in experiments, animals with visible health abnormalities were excluded. Mice were housed in cages holding an average of 3–4 mice per cage with a temperature-controlled environment and 12-h light/dark cycles. The animals had access to food and water ad libitum. All animal experiments were approved by the University of Pittsburgh’s Institutional Animal Care and Use Committee. To assess biodistribution of systemically injected EVs in aged mice, EVs were labeled with PKH26-dyed and imaged by LI-COR at 48 hours after the systemic injection.

### Human chondrocytes culture

This study followed the BRISQ reporting guideline^60^. Primary human chondrocytes from heathy older male donors (68 years old) were obtained through the StemBioSys company (San Antonio, TX) with approval from the University of Pittsburgh Committee for Oversight of Research and Clinical Training Involving Decedents (CORID). Isolated chondrocytes were seeded in tissue-culture flasks at a density of 0.5 × 10^4^ cells/cm^2^ and maintained in low-glucose DMEM supplemented with 15% FBS, 1% Glutamax, and 1% Pen/Strep at 37°C at 5% CO_2_. After cells were fully adhered to the culture substrate, the medium was changed every three days until cells reached 70–80% confluency. The cells were then detached with Trypsin/EDTA (Gibco/Thermo Fisher Scientific) and passaged. For all experiment, second passage chondrocytes were used.

### Aerobic exercise cohort

The aerobic exercise cohort was recruited from community-wide efforts, with the primary recruitment method being a clinical registry of individuals who expressed interest in engaging in this study. Individuals who expressed interest completed a telephone screening to determine initial eligibility. Individuals who appeared to be eligible attended a study orientation session where additional details of the study were provided, and written informed consent was obtained prior to proceeding with additional study procedures. Upon obtainment of informed consent, participants completed additional eligibility screening procedures and other baseline assessment measures. Participants who remained eligible were assigned to participate in the intervention, with one of the intervention conditions being aerobic exercise.

The aerobic exercise intervention paradigm consisted of a combination of 1 supervised session and 4 unsupervised (home-based) sessions per week. The duration of each of the supervised sessions was 30 minutes and the unsupervised sessions progressed from 10 minutes per session during weeks 1–4 to 20 minutes per session during weeks 5–8 to 30 minutes per session during weeks 9–12. Thus, the weekly duration of aerobic exercise progressed from 70 to 150 minutes per week across this period. The aerobic exercise was designed to elicit a moderate-intensity. To facilitate this, aerobic exercise videos were developed by the study staff that we used to guide the supervised exercise sessions and were also placed on tablets provided to the participants to facilitate the unsupervised, home-based exercise sessions.

### Serum collection

Blood was collected via venapuncture by a trained phlebotomist. Participants were instructed to fast for a period of 10–12 hours, except for water, and to also avoid strenuous physical activity for 10–12 hours prior to blood collection. Blood underwent initial processing, which included appropriate centrifuging and pipetting into 1 ml cryotubes prior to storage at −80°C until used for analysis. Samples displaying hemolysis (as evidenced by pink/red coloration) were not included in the analysis.

### EV isolation and characterization

EVs were isolated from serum using size-exclusion chromatography (cat no, SP6, qEVsingle 35-nm iZON columns) according to the manufacturer’s protocol and as we described^41^. Briefly, after single wash of column by 1mL PBS, 100 μL of serum was added onto the top filter of the column. The eluted volume was collected in small Eppendorf centrifuge tubes. The first five fractions (in total 1,000 μL) were collected in a 1.5 mL tube. These fractions contain minimal amount of EVs and are considered to be void fractions according to manufacturer’s guidelines. The majority of the EVs were eluted in fractions 6–11 (200 μL each). These fractions, a total of 1.2 mL, were collected together in a 2 mL tube. After isolation, EVs were stored at −20°C.

### Nanoparticle tracking analysis of EVs

The collected EVs were characterized for size and concentration by Nanoparticle tracking analysis (NTA) on a NanoSight NS300 (Malvern Panalytical). Ten μL from each EV sample was diluted 1:100 in EV-free water and infused through the flow-cell using a syringe pump (Harvard Apparatus 98–4730). Videos were recorded for three times in each sample, with the camera level set to 14. These videos were batch analyzed by the software (NTA 3.3) with the detection threshold set to 3. The flow-cell was washed with 1 mL of EV-free water between each sample.

### In-well Western for EV purity

A in-well western was performed to confirm EV purity. Briefly, 1×10^6^ EVs were fixed in 2% PFA for 10 minutes, followed by washing with PBS once. EVs were then blocked in PBS solution containing 3% BSA for 1 h at room temperature. After washing twice with PBS solution, EVs were incubated with Alex Fluor 647-labeled CD81(1:200, ThermoFisher) and GM130 (1: 200, Santa Cruz) overnight at 4°C, followed by secondary antibody incubation for 1 h at room temperature. EVs were washed twice with PBS, resuspended in 150 μL of PBS, and loaded to a 96-well plate. The fluorescent imaging was performed using LI-COR ODYSSEY CLx and LI-COR Image Studio Acquisition Software (LI-COR Biosciences, NE). Mouse primary muscle fibroblasts were used as the positive control for GM130.

### Preparation of fibronectin-coated pAAm substrates

We prepared pAAm gels with different stiffness (5 kPa and 100 kPa) in accordance with a previous study^61^. The pAAm gels were made on glass coverslips pre-treated with 0.1 N sodium hydroxide (cat. SS255–1, Fisher Scientific, IL), 0.5% 3-aminopropyltrimethoxysilane (cat no. AC313251000, Acros Organics, Belgium) and 0.5% glutaraldehyde (cat no. BP25481, Fisher Scientific, IL) to improve gel adhesion. To facilitate cell adhesion, the surfaces of prepared hydrogels were further conjugated with fibronectin (100 μg/mL, from bovine plasma, Sigma) using sulfo-SANPAH (cat no. NC1314883, Proteochem Inc., UT) as a crosslinker. Prior to seeding cells, gels were UV-sterilized in a cell culture hood for 30 minutes. Gels were kept hydrated in HEPES or PBS during all preparation steps.

### Evaluation of the impact of matrix stiffness on cell fate

Isolated primary chondrocytes from eldery were plated (5,000 cells per cm^2^) on 5kPa and 100kPa pAAm gels and cultured in low-glucose DMEM supplemented with 15% FBS, 1% Glutamax, and 1% Pen/Strep at 37°C at 5% CO_2_. On day 5, the culture medium was removed and seeded pAAm gels were fixed in 2% PFA for 10 minutes. After a triple wash by PBS, cells were kept in PBS at 4°C until used.

### Assessment of therapeutic potential of EVs

To test the ability of circulating EVs isolated before and after exercise to modulate chondrocytes health, the isolated EVs were administrated (0.5×10^9^ particles) on elderly human chondrocytes cultured on aged-like stiff pAAm substrate on day 3 after chondrocyte seeding. After additional 2-day culture with EVs (i.e., on day 5 after chondrocyte seeding), culture medium was removed and seeded pAAm gels were fixed in 2% PFA for 10 minutes. After a triple wash by PBS, cells were kept in PBS at 4°C until used.

### EV uptake by chondrocytes

EVs (1.0×10^9^ particles) isolated after exercise were labelled with PKH26 (cat no. PKH26GL-1KT, Sigma) according to the manufacturer’s instructions. Briefly, EVs were incubated with PKH26 for 10 minutes at room temperature. EVs were then washed in wash buffer and centrifuged at 16,100 g for 30 minutes at 4°C. The supernatant was removed until the 100 μL mark and the residual volume was mixed gently. Subsequently, the PKH26-labeled EVs were supplemented to human chondrocytes and incubated for 2 hours under live cell imaging (cat no. CX51110, Cellinsight CX5 HCS Platform, ThermoFisher). Chondrocytes were visualized using MitoTracker.

### Immunofluorescence and imaging

Immunofluorescence analysis for cell-seeded pAAm substrate was performed to determine the signal intensity of type II collagen, Sox9, and DNMT1 in accordance with established protocols^62^. Briefly, after a triple wash by PBS, cells were permeabilized with 0.1% triton-X (Fluka 93420) for 15 minutes, followed by blocking for 1 hour in 0.1% triton-X and 3% Bovine Serum Albumin (BSA, Sigma A7906) in PBS. After blocking, the cells were incubated overnight at 4°C with primary antibodies for type II collagen (1:200; Ab34712, Abcam), Sox9 (1:200; #82630, Cell Signaling), DNMT1 (1:200; #5032, Cell Signaling) in antibody solution (0.1% Triton-X + 3% BSA + 5% Goat Serum). One negative control slide per staining set was generated by omitting the primary antibody in the antibody solution.

After a triple wash by PBS, the samples were incubated with host-specific secondary antibodies conjugated with Alexa Fluor 488 (Fisher Scientific) in antibody solution for one hour at room temperature at a 1:200 dilution. Following a triple wash with PBS, the samples were stained with DAPI for 2 minutes and then washed with PBS again. Samples were mounted with coverslips using Gelvatol mounting medium (Source: Center of Biologic Imaging, University of Pittsburgh). Slides were imaged using a Zeiss Observer Z1 semi-confocal microscope with ZEN 2.3 software (Zeiss, Jena, Germany). All images were collected at 20x (type II collagen) or 63x (Sox9 and DNMT1) magnification. Negative control slides were used to the threshold for the signal intensity and to set the exposure time for individual channels. All images for quantitative analysis in a given experiment were taken under the same imaging conditions. Fluorescence intensity was quantified using Image J, in which integrated density was divided by number of cells in each image. We took 5–10 random images in each sample and averaged them for statistical analysis.

### Quantification of cellular morphology

F-actin images were obtained at 20x magnification using a Zeiss Observer Z1 semi-confocal microscope with ZEN 2.3 software (Zeiss, Jena, Germany). Image processing and morphological feature extraction were performed using CellProfiler software (v4.0, The Broad Institute)^44^. Fifty-three shape features of chondrocytes were determined using the “identify primary objects” followed by the “measure object size shape” and “export to spreadsheet” modules.

### Unsupervised machine learning

PCA was performed for data reduction to identify the principal components that represent differences in the cellular morphology. To determine variables of cellular shape contributing to principal components, the loading matrix, a correlation between the original variables and PCs, was extracted. In addition, PCA was used to visualize the separation of (1) chondrocytes cultured on soft and stiff pAAm substrates, and (2) chondrocytes cultured on stiff pAAm supplemented with EV^pre^ and EV^post^.

### Global DNA methylation assay

Levels of 5 methylcytosine (5mC) in DNA were measured using the 5mC DNA ELISA Kit (Zymo Research, Irvine, CA). A total of 200 ng of DNA was used and the percent of 5mC in the samples was quantified, normalized to total DNA, and compared to the standard curve provided in the kit.

### Real time-PCR

500ng of isolated RNA was reverse transcribed using iScript^™^ Advanced cDNA Synthesis Kit (BIO-RAD, Hercules, CA). The mRNA levels of the Klotho gene were quantified by SYBR Green-based real-time PCR (qPCR) using SsoAdvanced^™^ Universal SYBR^®^ Green Supermix (BIO-RAD, Hercules, CA). *Klotho* gene expression levels were normalized to the expression level of *RPL19*, and the fold change of *Klotho* relative to universal human reference RNA was calculated using 2-ΔΔCt method. Primer sequences: Klotho (NM_004795.4) Fwd- 5’-CAAGTACTGGATCACCATCGACA-3’ and Rev-5’-AGACTTTGGCATGAGCCAGGAG-3’. RPL19 (NM_000981) Fwd-5′-AAGCCTGTGACGGTCCATTC-3’ and Rev-5’-CCCTTCCGCTTACCTATGCC-3’.

### Methylation-specific PCR

200 ng of genomic DNA was subjected to bisulfite conversion with an EZ DNA Methylation Kit (Zymo Research, Irvine, CA) followed by methylation-specific PCR (MSPCR) using primers specific for methylated DNA of Klotho promoter. Differentially methylated region of human Klotho promoter in 5-aza-dC treated cells was reported (PMID: 22237753). Primer sequences were designed to amplify the region of −306 to −89 (NG_011485.1:4685–4902). hMS-Kl-Fwd: 5’- AAAGAGAATGAATTTGAGCGTTTAC – 3’ and hMS-Kl-Rev: 5’- ACTCCGCTAACAATAATTACCTACG – 3’. Fully methylated control DNA (Zymo Research, Irvine, CA) was used as a reference to calculate the percentage methylation of DNA samples.

### Statistical analysis

All statistical analyses were performed using JMP Pro 14 software (SAS Institute, Cary, NC). Except where indicated, data are displayed as means, with uncertainty expressed as 95% confidence intervals. For unpaired experiments, two-tailed Student *t*-test was performed. For paired experiments, two-tailed paired *t*-test was utilized. Linear regression analysis was performed to assess the relationship among cellular morphological variable, type II collagen expression, and Sox9 expression. We checked the features of the regression model by comparing the residuals vs. fitted values (i.e., the residuals had to be normally distributed around zero) and independence between observations. No correction was applied for multiple comparison because outcomes were determined *priori* and were highly correlated. In all experiments, p-values < 0.05 were considered statistically significant. Except where indicated, throughout this text, “*n*” represents the number of independent observations. Specific data representation details and statistical procedures are also indicated in the figure legends.

## Data Availability

The raw data that support the experimental findings are included in article supplementary material. Any additional information required to reanalyze the data reported in this work is available from the corresponding author upon request.

## References

[1] Denham J. & Spencer S.J. Emerging roles of extracellular vesicles in the intercellular communication for exercise-induced adaptations. Am J Physiol Endocrinol Metab 319, E320–e329 (2020). 32603601 10.1152/ajpendo.00215.2020

[2] Zaborowski M.P., Balaj L., Breakefield X.O. & Lai C.P. Extracellular vesicles: composition, biological relevance, and methods of study. Bioscience 65, 783–797 (2015). 26955082 10.1093/biosci/biv084 PMC4776721

[3] Nair V.D., Sedentary and Trained Older Men Have Distinct Circulating Exosomal microRNA Profiles at Baseline and in Response to Acute Exercise. Front Physiol 11, 605 (2020). 32587527 10.3389/fphys.2020.00605 PMC7298138

[4] Vanderboom P.M., A size-exclusion-based approach for purifying extracellular vesicles from human plasma. Cell Rep Methods 1(2021). 10.1016/j.crmeth.2021.100055 PMC8336930 34355211

[5] Bei Y., Exercise-induced circulating extracellular vesicles protect against cardiac ischemia-reperfusion injury. Basic Res Cardiol 112, 38 (2017). 28534118 10.1007/s00395-017-0628-z PMC5748384

[6] Safdar A., Saleem A. & Tarnopolsky M.A. The potential of endurance exercise-derived exosomes to treat metabolic diseases. Nat Rev Endocrinol 12, 504–517 (2016). 27230949 10.1038/nrendo.2016.76

[7] Pegtel D.M., Functional delivery of viral miRNAs via exosomes. Proc Natl Acad Sci U S A 107, 6328–6333 (2010). 20304794 10.1073/pnas.0914843107 PMC2851954

[8] Herranz H. & Cohen S.M. MicroRNAs and gene regulatory networks: managing the impact of noise in biological systems. Genes Dev 24, 1339–1344 (2010). 20595229 10.1101/gad.1937010 PMC2895193

[9] Arner P. & Kulyté A. MicroRNA regulatory networks in human adipose tissue and obesity. Nat Rev Endocrinol 11, 276–288 (2015). 25732520 10.1038/nrendo.2015.25

[10] Wong A.K., Krishnan A. & Troyanskaya O.G. GIANT 2.0: genome-scale integrated analysis of gene networks in tissues. Nucleic Acids Res 46, W65–w70 (2018). 29800226 10.1093/nar/gky408 PMC6030827

[11] Lage K., A large-scale analysis of tissue-specific pathology and gene expression of human disease genes and complexes. Proc Natl Acad Sci U S A 105, 20870–20875 (2008). 19104045 10.1073/pnas.0810772105 PMC2606902

[12] McAlindon T.E., OARSI guidelines for the non-surgical management of knee osteoarthritis. Osteoarthritis and cartilage 22, 363–388 (2014). 24462672 10.1016/j.joca.2014.01.003

[13] Chen Y., Lun A.T. & Smyth G.K. From reads to genes to pathways: differential expression analysis of RNA-Seq experiments using Rsubread and the edgeR quasi-likelihood pipeline. F1000Res 5, 1438 (2016). 27508061 10.12688/f1000research.8987.1 PMC4934518

[14] Kriegel A.J., Liu Y., Fang Y., Ding X. & Liang M. The miR-29 family: genomics, cell biology, and relevance to renal and cardiovascular injury. Physiol Genomics 44, 237–244 (2012). 22214600 10.1152/physiolgenomics.00141.2011 PMC3289120

[15] Liu Y., Renal medullary microRNAs in Dahl salt-sensitive rats: miR-29b regulates several collagens and related genes. Hypertension 55, 974–982 (2010). 20194304 10.1161/HYPERTENSIONAHA.109.144428 PMC2862728

[16] Ramdas V., McBride M., Denby L. & Baker A.H. Canonical transforming growth factor-β signaling regulates disintegrin metalloprotease expression in experimental renal fibrosis via miR-29. Am J Pathol 183, 1885–1896 (2013). 24103556 10.1016/j.ajpath.2013.08.027 PMC4188136

[17] Cheng J., Wang Y., Wang D. & Wu Y. Identification of collagen 1 as a post-transcriptional target of miR-29b in skin fibroblasts: therapeutic implication for scar reduction. Am J Med Sci 346, 98–103 (2013). 23221517 10.1097/MAJ.0b013e318267680d

[18] Montgomery R.L., MicroRNA mimicry blocks pulmonary fibrosis. EMBO Mol Med 6, 1347–1356 (2014). 25239947 10.15252/emmm.201303604 PMC4287936

[19] Ko J.-Y., MicroRNA-29a counteracts synovitis in knee osteoarthritis pathogenesis by targeting VEGF. Scientific reports 7, 1–14 (2017). 28620193 10.1038/s41598-017-03616-w PMC5472675

[20] Kawanishi N., Exercise training attenuates hepatic inflammation, fibrosis and macrophage infiltration during diet induced-obesity in mice. Brain, behavior, and immunity 26, 931–941 (2012). 22554494 10.1016/j.bbi.2012.04.006

[21] Kern F., miEAA 2.0: integrating multi-species microRNA enrichment analysis and workflow management systems. Nucleic Acids Res 48, W521–w528 (2020). 32374865 10.1093/nar/gkaa309 PMC7319446

[22] Huang H.-Y., miRTarBase 2020: updates to the experimentally validated microRNA–target interaction database. Nucleic acids research 48, D148–D154 (2020). 31647101 10.1093/nar/gkz896 PMC7145596

[23] Berrier A.L. & Yamada K.M. Cell-matrix adhesion. J Cell Physiol 213, 565–573 (2007). 17680633 10.1002/jcp.21237

[24] Greene C.S., Understanding multicellular function and disease with human tissue-specific networks. Nat Genet 47, 569–576 (2015). 25915600 10.1038/ng.3259 PMC4828725

[25] Reverter A., Ingham A. & Dalrymple B.P. Mining tissue specificity, gene connectivity and disease association to reveal a set of genes that modify the action of disease causing genes. BioData Min 1, 8 (2008). 18822114 10.1186/1756-0381-1-8 PMC2556670

[26] Zhao Z., Mechanotransduction pathways in the regulation of cartilage chondrocyte homoeostasis. Journal of cellular and molecular medicine 24, 5408–5419 (2020). 32237113 10.1111/jcmm.15204 PMC7214151

[27] Cowen L., Ideker T., Raphael B.J. & Sharan R. Network propagation: a universal amplifier of genetic associations. Nat Rev Genet 18, 551–562 (2017). 28607512 10.1038/nrg.2017.38

[28] Knight A. & Levick J.R. Morphometry of the ultrastructure of the blood-joint barrier in the rabbit knee. Quarterly Journal of Experimental Physiology: Translation and Integration 69, 271–288 (1984). 10.1113/expphysiol.1984.sp002805 6729017

[29] Valdeolivas A., Random walk with restart on multiplex and heterogeneous biological networks. Bioinformatics 35, 497–505 (2019). 30020411 10.1093/bioinformatics/bty637

[30] Kanehisa M., Sato Y., Kawashima M., Furumichi M. & Tanabe M. KEGG as a reference resource for gene and protein annotation. Nucleic Acids Res 44, D457–462 (2016). 26476454 10.1093/nar/gkv1070 PMC4702792

[31] Yao Q., Osteoarthritis: pathogenic signaling pathways and therapeutic targets. Signal Transduction and Targeted Therapy 8, 56 (2023). 36737426 10.1038/s41392-023-01330-w PMC9898571

[32] Iijima H., Age-related matrix stiffening epigenetically regulates α-Klotho expression and compromises chondrocyte integrity. Nature Communications 14, 18 (2023). 10.1038/s41467-022-35359-2 PMC9832042 36627269

[33] Phillip J.M., Aifuwa I., Walston J. & Wirtz D. The mechanobiology of aging. Annual review of biomedical engineering 17, 113–141 (2015). 10.1146/annurev-bioeng-071114-040829 PMC4886230 26643020

[34] Schmauck-Medina T., New hallmarks of ageing: a 2022 Copenhagen ageing meeting summary. Aging (Albany NY) 14, 6829–6839 (2022). 36040386 10.18632/aging.204248 PMC9467401

[35] Stolz M., Early detection of aging cartilage and osteoarthritis in mice and patient samples using atomic force microscopy. Nat Nanotechnol 4, 186–192 (2009). 19265849 10.1038/nnano.2008.410

[36] Selman M. & Pardo A. Fibroageing: An ageing pathological feature driven by dysregulated extracellular matrix-cell mechanobiology. Ageing Res Rev 70, 101393 (2021). 34139337 10.1016/j.arr.2021.101393

[37] Kim J.H., Matrix cross-linking-mediated mechanotransduction promotes posttraumatic osteoarthritis. Proc Natl Acad Sci U S A 112, 9424–9429 (2015). 26170306 10.1073/pnas.1505700112 PMC4522801

[38] Zhong W., YAP-mediated regulation of the chondrogenic phenotype in response to matrix elasticity. J Mol Histol 44, 587–595 (2013). 23543231 10.1007/s10735-013-9502-y

[39] Du J., Extracellular matrix stiffness dictates Wnt expression through integrin pathway. Sci Rep 6, 20395 (2016). 26854061 10.1038/srep20395 PMC4745056

[40] Zhou C., Microenvironmental stiffness mediates cytoskeleton re-organization in chondrocytes through laminin-FAK mechanotransduction. Int J Oral Sci 14, 15 (2022). 35277477 10.1038/s41368-022-00165-5 PMC8917190

[41] Sahu A., Regulation of aged skeletal muscle regeneration by circulating extracellular vesicles. Nature Aging 1, 1148–1161 (2021). 35665306 10.1038/s43587-021-00143-2 PMC9165723

[42] Estébanez B., Jiménez-Pavón D., Huang C.J., Cuevas M.J. & González-Gallego J. Effects of exercise on exosome release and cargo in in vivo and ex vivo models: A systematic review. J Cell Physiol 236, 3336–3353 (2021). 33037627 10.1002/jcp.30094

[43] Chong M.C., Silva A., James P.F., Wu S.S.X. & Howitt J. Exercise increases the release of NAMPT in extracellular vesicles and alters NAD(+) activity in recipient cells. Aging Cell 21, e13647 (2022). 35661560 10.1111/acel.13647 PMC9282849

[44] Carpenter A.E., CellProfiler: image analysis software for identifying and quantifying cell phenotypes. Genome Biol 7, R100 (2006). 17076895 10.1186/gb-2006-7-10-r100 PMC1794559

[45] Moore L.D., Le T. & Fan G. DNA methylation and its basic function. Neuropsychopharmacology 38, 23–38 (2013). 22781841 10.1038/npp.2012.112 PMC3521964

[46] Li X., Zhen Z., Tang G., Zheng C. & Yang G. MiR-29a and MiR-140 Protect Chondrocytes against the Anti-Proliferation and Cell Matrix Signaling Changes by IL-1β. Mol Cells 39, 103–110 (2016). 26608362 10.14348/molcells.2016.2179 PMC4757797

[47] Sun K., The PI3K/AKT/mTOR signaling pathway in osteoarthritis: a narrative review. Osteoarthritis Cartilage 28, 400–409 (2020). 32081707 10.1016/j.joca.2020.02.027

[48] Liu S., PI3K/Akt inhibitor partly decreases TNF-α-induced activation of fibroblast-like synoviocytes in osteoarthritis. Journal of orthopaedic surgery and research 14, 425 (2019). 31829201 10.1186/s13018-019-1394-4 PMC6907257

[49] Jiang R.H., Glycyrrhizin inhibits osteoarthritis development through suppressing the PI3K/AKT/NF-κB signaling pathway in vivo and in vitro. Food & function 11, 2126–2136 (2020). 32073014 10.1039/c9fo02241d

[50] Lampi M.C. & Reinhart-King C.A. Targeting extracellular matrix stiffness to attenuate disease: From molecular mechanisms to clinical trials. Sci Transl Med 10(2018). 10.1126/scitranslmed.aao0475 29298864

[51] Guo S., Stimulating Extracellular Vesicles Production from Engineered Tissues by Mechanical Forces. Nano Letters 21, 2497–2504 (2021). 33709717 10.1021/acs.nanolett.0c04834

[52] Najrana T., Mechanical stretch regulates the expression of specific miRNA in extracellular vesicles released from lung epithelial cells. J Cell Physiol 235, 8210–8223 (2020). 31970782 10.1002/jcp.29476 PMC7374064

[53] Percie du Sert N., The ARRIVE guidelines 2.0: Updated guidelines for reporting animal research. PLoS biology 18, e3000410 (2020). 32663219 10.1371/journal.pbio.3000410 PMC7360023

[54] Emmerich C.H. & Harris C.M. Minimum information and quality standards for conducting, reporting, and organizing in vitro research. Bespalov A., Michel M., and Steckler T. Cham: Springer 257, 177–196 (2019). 10.1007/164_2019_284 31628600

[55] Dupont W.D. & Plummer W.D. Jr. Power and sample size calculations for studies involving linear regression. Control Clin Trials 19, 589–601 (1998). 9875838 10.1016/s0197-2456(98)00037-3

[56] Vechetti I.J. Jr., Valentino T., Mobley C.B. & McCarthy J.J. The role of extracellular vesicles in skeletal muscle and systematic adaptation to exercise. J Physiol 599, 845–861 (2021). 31944292 10.1113/JP278929 PMC7363579

[57] Safdar A. & Tarnopolsky M.A. Exosomes as Mediators of the Systemic Adaptations to Endurance Exercise. Cold Spring Harb Perspect Med 8(2018). 10.1101/cshperspect.a029827 PMC5830902 28490541

[58] Robinson M.D., McCarthy D.J. & Smyth G.K. edgeR: a Bioconductor package for differential expression analysis of digital gene expression data. bioinformatics 26, 139–140 (2010). 19910308 10.1093/bioinformatics/btp616 PMC2796818

[59] Ritchie M.E., limma powers differential expression analyses for RNA-sequencing and microarray studies. Nucleic acids research 43, e47–e47 (2015). 25605792 10.1093/nar/gkv007 PMC4402510

[60] Moore H.M., Biospecimen reporting for improved study quality (BRISQ). Journal of proteome research 10, 3429–3438 (2011). 21574648 10.1021/pr200021n PMC3169291

[61] Wang K., Substrate Stiffness-Dependent Carbon Nanotube-Induced Lung Fibrogenesis. Nano letters 19, 5443–5451 (2019). 31369708 10.1021/acs.nanolett.9b01943 PMC6724206

[62] Sahu A., Age-related declines in α-Klotho drive progenitor cell mitochondrial dysfunction and impaired muscle regeneration. Nat Commun 9, 4859 (2018). 30451844 10.1038/s41467-018-07253-3 PMC6242898

